# A Roadmap for Understanding Memory: Decomposing Cognitive Processes into Operations and Representations

**DOI:** 10.1523/ENEURO.0122-19.2019

**Published:** 2019-07-08

**Authors:** Rosemary A. Cowell, Morgan D. Barense, Patrick S. Sadil

**Affiliations:** 1 Department of Psychological and Brain Sciences, University of Massachusetts Amherst, Amherst, Massachusetts 01003; 2 Department of Psychology, University of Toronto, Toronto, Ontario M5S 3G3, Canada

**Keywords:** cognitive process, familiarity, memory, pattern completion, recollection, representation

## Abstract

Thanks to patients Phineas Gage and Henry Molaison, we have long known that behavioral control depends on the frontal lobes, whereas declarative memory depends on the medial temporal lobes (MTL). For decades, cognitive functions—behavioral control, declarative memory—have served as labels for characterizing the division of labor in cortex. This approach has made enormous contributions to understanding how the brain enables the mind, providing a systems-level explanation of brain function that constrains lower-level investigations of neural mechanism. Today, the approach has evolved such that functional labels are often applied to brain networks rather than focal brain regions. Furthermore, the labels have diversified to include both broadly-defined cognitive functions (declarative memory, visual perception) and more circumscribed mental processes (recollection, familiarity, priming). We ask whether a process—a high-level mental phenomenon corresponding to an introspectively-identifiable cognitive event—is the most productive label for dissecting memory. For example, recollection conflates a neurocomputational *operation* (pattern completion-based retrieval) with a class of *representational content* (associative, high-dimensional memories). Because a full theory of memory must identify operations and representations separately, and specify how they interact, we argue that processes like recollection constitute inadequate labels for characterizing neural mechanisms. Instead, we advocate considering the component operations and representations of processes like recollection in isolation. For the organization of memory, the evidence suggests that pattern completion is recapitulated widely across the ventral visual stream and MTL, but the division of labor between sites within this pathway can be explained by representational content.

## Significance Statement

Accounts of cognition often assume that the brain is organized along lines of cognitive process, for example, with recollection mediated by one neural structure and familiarity by another. We argue that cognitive processes—introspectively-identifiable mental events like recollection—are inadequate labels for characterizing neural mechanisms, because they conflate lower-level components of the mechanisms we seek to identify. Recollection involves both a neurocomputational operation (pattern completion) and a neural representation (high-dimensional, associative content). To uncover memory's mechanisms, we must decompose memory processes into their operations and representations, asking how each contributes to mnemonic phenomena. Decomposing recollection suggests that, within the ventral visual pathway and MTL, different brain regions contribute to memory retrieval according to their representational content.

## Introduction

Since Phineas Gage and Patient H.M., we have known that distinct cognitive abilities can be mapped onto discrete brain regions: key aspects of behavioral control depend on the frontal lobes, whereas the ability to form long-term declarative memories depends on the medial temporal lobes (MTLs). The topological framework provided by such mappings offers valuable systems-level explanations of brain function that critically constrain lower-level investigations of neural mechanism. Thus, a major goal of cognitive neuroscience could be crudely characterized as a well-founded game of pin the tail on the donkey, in which “blindfolded” researchers pin cognitive functions to the cerebral cortex. This approach has made enormous contributions to understanding how the brain enables the mind. Today, the approach has evolved such that the functional labels are often applied to brain networks rather than focal brain regions. Furthermore, the labels describing the division of labor have diversified to include both broadly-defined cognitive functions (declarative memory vs visual perception, short-term vs long-term memory) and more circumscribed mental processes (recollection versus familiarity). In this review, we take *recollection* as an example, asking whether this process-based account provides the best description of the available data concerning how different brain regions contribute to declarative memory. If not, what alternative set of labels could be used to capture the organization of memory retrieval in the ventral visual pathway and MTL?

## What is recollection?

Recollection has a long history as an explanatory construct in experimental and cognitive psychology. A recollection-like process was described in detail by William James in 1890, and has since been invoked by many authors in various guises—an intentional search of memory; the effortful retrieval of context and detail; autonoetically-conscious remembering (James, 1890; Hasher and Zacks, 1979; Tulving, 1985; Jacoby, 1991). In modern memory theories, recollection is typically defined as a pattern completion-like retrieval mechanism, in which a cue (external or internal) prompts the conscious remembering of specific details of an episode (Marr, 1971; Tulving, 1982; Rolls, 1996; Norman and O’Reilly, 2003; Mayes et al., 2007). Here, an episode is some unique prior event defined by arbitrarily associated elements, and the remembered details are present at the time of encoding but absent at the time of retrieval. Some have argued for implicit influences on, or components of, recollection (Turk-Browne et al., 2006; Sheldon and Moscovitch, 2010; Taylor and Henson, 2012; Taylor et al., 2013). We do not contest these claims, but here we focus on the canonical aspects of recollection in which the remembered details are brought to mind explicitly.

Recollection is frequently contrasted with a less precise, more automatic signal-strength process, often termed familiarity (Mandler et al., 1969; Mandler, 1980; Juola et al., 1971; Anderson and Bower, 1972; Atkinson and Juola, 1973; Tulving, 1985). Familiarity—the subjective sense that an item or situation has been encountered before—has been proposed to depend on a continuous memory strength signal (Kintsch, 1967; Juola et al., 1971). The value of this signal for a given item is augmented by learning when the item is encountered. Familiarity therefore provides a basis for judging prior occurrence, because previously encountered items on average possess higher signal strength than novel items. The contrast between recollection and familiarity is a popular process-based distinction in memory research. For example, “dual-process” theories of recognition memory assume that recollection and familiarity jointly underlie judgements of prior occurrence, and have inspired empirical frameworks for dissociating the relative contributions of the two processes (Jacoby, 1991; Yonelinas, 1994; Yonelinas and Jacoby, 1994). Although some recent studies have questioned the strength of the evidence for dual-process models (Wixted, 2007; Dunn, 2008; Ratcliff and Starns, 2009; Jang et al., 2011; Rotello, 2017), the dual-process framework has been an influential and successful approach within cognitive neuroscience for explaining how distinct brain regions contribute to remembering (Aggleton and Brown, 1999, 2006; Brown and Aggleton, 2001; Meeter et al., 2005; Eichenbaum et al., 2007; Montaldi and Mayes, 2010; Webster et al., 2013; Wang et al., 2018). In these models, which emphasize the well-documented role of MTL structures in declarative memory, recollection is typically ascribed to the hippocampus, or a network including the hippocampus, whereas familiarity is associated with distinct neocortical sites such perirhinal cortex.

Here, we set aside the debate over whether recognition memory draws on one or two processes [for a discussion of this, see Rotello (2017) or a special issue introduced by Voss and Paller (2010)], and simply assume that a recollection-like process is implemented by the brain in at least some memory tasks, such as cued recall. Our goal is to ask whether process labels like “recollection” are useful for explaining the contributions of distinct regions within the ventral visual pathway and MTL to memory. Or would alternative labels, based on something other than mnemonic processes, do a better job?

## Decomposing cognitive processes into operations and representations

To ask whether cognitive process labels provide an adequate account of the neuroanatomical organization of memory retrieval, we must first define what we mean by a “cognitive process”. Although process-to-brain mappings are made frequently in cognitive neuroscience, a cognitive process can mean different things to different researchers. We define a *cognitive process* as a mental or behavioral phenomenon corresponding to an introspectively-identifiable cognitive event; it always involves some cognitive or neural operation, but it may or may not be constrained to apply to a particular type of representation. In this definition, an *operation* is an algorithmic computation performed by the brain, e.g., pattern completion, generation of a neural signal corresponding to memory strength, or computation of a match between two signals; in contrast, a *representation* is a pattern of neural firing that stands in for an event, stimulus, or stimulus attribute in the world, e.g., the color red, or a multimodal association of complex elements corresponding to an episodic event (Cowell, 2012). The following all constitute examples of what we take as a cognitive process: recollection, conflict-monitoring, face recognition, syntactic parsing, phonological encoding, or inhibition of action (Mandler, 1980; Friederici et al., 1996; Botvinick et al., 2001; Aron and Poldrack, 2006; Chen et al., 2012). These processes comprise a diverse set of mental functions, but they have at least one thing in common: they correspond to an identifiable phenomenon of mental life or behavior; most adults know how it feels to recollect a memory, recognize a face, make grammatical sense (or struggle to make sense) of a spoken sentence, produce intended words from the correct phonemes (or fail to, as in “well-boiled icicle” for “well-oiled bicycle”), or release the accelerator when a speed camera is spotted on the road ahead.

But this definition of a cognitive process, which broadly corresponds to the use of the term in the literature, reveals a problem with using processes as components of a theory of brain function. Because processes are high-level behavioral phenomena, the mapping of process labels to neural structures in theories of cognition constitutes a category mistake: these concepts are the phenomena to be explained, not the components of an explanatory mechanism. Recollection and familiarity emerge from the representations and operations computed by neural structures, in the same way that team spirit emerges from the players on a cricket team (Ryle, 1949). If the goal of building a theory is to describe a set of behavioral phenomena in terms of the component neural structures, operations, and representations that give rise to them, those phenomena cannot serve as the components giving rise to themselves (Bechtel, 2008a). Instead, we need intermediate-level operations couched at a lower level than the phenomenon itself, e.g., pattern completion (see Box 1).

In some cases, a cognitive process may be reducible to an operation alone, for example, *judging familiarity* requires only generating a continuous memory strength signal and assessing its strength, with no stipulation about the representations that the operations act on. In such cases, we nonetheless advocate transforming the label from one that evokes a behavioral phenomenon to one that describes a neurocomputational operation, because the latter offers a more precise characterization that begins to specify lower-level mechanisms. However, a more problematic scenario is when a cognitive process label (often implicitly) encompasses both an operation and representation, conflating these two components of neurocognitive mechanism. Because any explanatory theory of brain function must ultimately specify both components separately, an important question is whether one or other alone can explain how different brain regions contribute to memory. If the labels we map onto the brain are hybrid processes that blend operation with representation, this question will remain unanswered.

Recollection is a case in point. Because recollection is usually said to entail retrieving specific details, it almost always requires that the representation supporting retrieval contains a unique, arbitrary association, for example, between an item and its environmental context, an image and a thought, or an event and a temporal context. In other words, because recollection specifies the kind of information that must be retrieved, it is a hybrid process: it encompasses both an operation—pattern completion-based retrieval (and possibly others, but we focus on this key operation as a test case)—and a class of representational content—memory for unique, arbitrary associations between relatively complex elements (e.g., aspects of the study episode, contextual information regarding the encoding context). Therefore, if we wish to elucidate both how the brain implements retrieval operations and how it represents memories, searching for the cortical locus of recollection will not serve us well. We need to decompose recollection into a *content-neutral retrieval operation* and a separate definition of the *representational content* on which retrieval operates. By separating these components, we can consider the influence of each in isolation on the engagement of various brain structures. Then we might discover whether a single component—representational content or retrieval operation—can account for the neuroanatomical organization of memory retrieval on its own, or whether both confer explanatory power.

Before proceeding, we clarify an important point: a full theory of declarative memory requires both representations and operations. Even if one of these alone turns out to account for how brain structures contribute to memory, we cannot discard the other. If brain regions contribute according to the operation they perform, a theory must still specify how representations are constructed (although representations are not constrained by location in the brain). If brain regions contribute according to the representations they contain, a theory must still specify how operations are performed (although an operation may unfold in many cortical sites). Thus, our central question is: how is the brain carved up in terms of its contributions to memory? This information is an important precursor to a complete theory of memory.

## Box 1. Banishing ghosts of process from the neural machinery of human memory

Many theories of memory are based on the premise that introspectively-identifiable processes such as recollection and familiarity can explain, in whole or in part, the contributions of distinct brain regions to memory retrieval. According to philosophers who have studied explanations in modern neuroscience and psychology (Bechtel, 2008a; Kaplan and Craver, 2011; Weiskopf, 2011), this follows a popular scientific practice: scientists in all fields seek mechanistic explanations of phenomena in which “a structure perform[s] a function in virtue of its component parts, component operations, and their organization” (Bechtel, 2008a). Thus, the mapping of recollection and familiarity onto hippocampus and nearby neocortex, respectively, appears to provide a good example of a mechanistic explanation. If our target phenomenon is “the brain-basis of declarative memory performance”, then these theories explain it by carving it into component processes and mapping them onto component parts of the brain.

However, to specify a mechanism, a phenomenon must be broken down into intermediate-level components: structures and functions corresponding to smaller-scale parts and more precise, well-specified operations (Bechtel, 2005, 2008a). To illustrate the idea of intermediate-level components, Bechtel has used the example of 19th century scientists seeking a mechanistic account of fermentation. Chemists focused on the atoms that make up sugar and alcohol; in doing so, they invoked components at too low a level, for this left open too many possible ways in which sugar could undergo reconfiguration to produce alcohol, thus failing to constrain the search for the correct mechanism. In contrast, physiologists attempted to identify intermediate components in the reaction—fragments of molecules larger than single atoms—but they referred to the operations on the fragments as “fermentation”; these accounts invoked operations at too high a level, for they described the operations giving rise to the phenomenon in terms of the phenomenon itself. It was eventually determined that fermentation occurs via addition, deletion or creation of particular *functional groups* of atoms, such as hydroxyl (-OH), to carbon-based substrate molecules. Only once these intermediate-level components and operations were identified was a mechanistic explanation attained (Bechtel, 2008a,b).

We suggest that, for theories that map recollection and familiarity onto, e.g., hippocampus and neocortex, the brain structures constitute intermediate-level parts, but the processes are not intermediate-level operations. Recollection and familiarity are subtypes of memory retrieval, not component operations. They characterize memory performance in greater phenomenological detail, arguably supplying an important precursor to an explanation (Bechtel, 2008a), but they provide no information about how the underlying cognitive operations are conducted. As in the case of fermentation, to ascribe recollection and familiarity to the brain’s component parts is to take high-level descriptions of the phenomenon we wish to explain and reapply them to smaller structural parts. In other scientific fields, explanatory breakthroughs have proven scant until the proper intermediate-level operations are identified (Bechtel, 2005).

Thus, we argue that processes are the high-level phenomena to be explained, and to use them as intermediate-level components in an account of memory performance is to make a category mistake. Recollection and familiarity are realized via the representations and operations computed by neural structures, in the same way that a university is realized through the existence and interactions of its libraries, science laboratories, administrative offices, students, staff, and faculty: no one component “is” the university (Ryle, 1949). In seeking intermediate-level components, we should instead use cognitive operations, such as pattern completion, and properties of stimulus representations, such as dimensionality. Operations and representations are intermediate because they supply information about how the neural substrates do their job. For example, the label “feature representation” implies a neural code richer in feature-based information than in information about whole objects. This can be mathematically defined, implemented in a computational model (Bussey and Saksida, 2002; Cowell et al., 2006, 2010a) and measured empirically (Cowell et al., 2017), and it has consequences for the outputs of that cortical region. This label thereby begins to deconstruct and specify the mechanisms of cognition. A further advantage of representational labels is that, unlike a process such as recollection, they do not appear to explain more than they do, because they make clear what is left to be specified; namely, the operations that act on the representations to produce a behavioral output, e.g., recall of a memory. A further advantage of operation labels (e.g., pattern completion) is that they can prompt the drawing of mechanistic parallels between two high-level phenomena, if a single operation contributes to two phenomena (e.g., recollection and priming; Sadil and Cowell, 2017).

To provide a full explanation of memory performance, both operations and representations must ultimately be specified, along with an account of how they interact. But, regardless of whether operations or representations turn out to provide a better account of the organization of memory in the ventral pathway (see Fig. 2), the task of discovering the full mechanism will be easier having first established the correct neuroanatomical constraints. Most importantly, these labels inch us closer to identifying the intermediate-level components by which a cognitive process such as recollection is realized. Once we have done so, the process itself may appear to be a “ghost in the machine” (Ryle, 1949); a phenomenon to be explained rather than a mechanistic component of a memory theory. By banishing these ghosts from the mechanisms we propose, we might arrive at an account of memory performance that is both more parsimonious and more accurate.

### Decomposing recollection: what is the operation?

We define recollection’s retrieval operation by adopting the mechanism typically assumed; a pattern completion-like operation. We define *pattern completion-like* as retrieval that is initiated by a partial cue (e.g., provided by an experimenter, a context, or a thought) and ends with the reinstatement of information that was stored with the cue but is not present in the current environment. This definition assumes nothing about the content of the memory representation on which the operation is conducted (Taylor and Henson, 2012).

There are, of course, other operations that might contribute to recollection, and certainly to memory retrieval more broadly, such as prediction errors, expectancy violations, and familiarity/novelty detection (Xiang and Brown, 1998; O’Connor et al., 2010; Exton-McGuinness et al., 2015). We focus on pattern completion because we believe it is central to recollection, and because it can be defined in a relatively straightforward manner, enabling its use as a test case.

### Decomposing recollection: what is the representational content?

**Figure 1.**
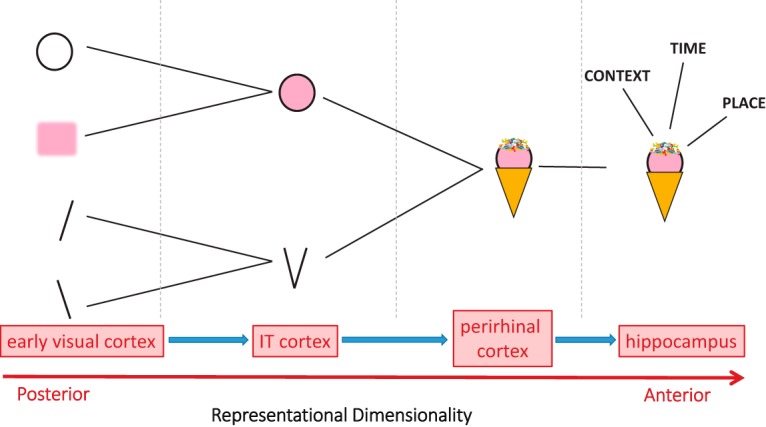
A representational-hierarchical account of cognition. Throughout the ventral stream and medial temporal lobe, stimulus features, objects, and events are represented as conjunctions of increasing dimensionality. IT, Inferotemporal. Each station in the hierarchy is engaged in the formation and retrieval of a memory to the extent that it represents the content of that memory. Particular operations (e.g., pattern completion-like retrieval, generation/readout of a memory strength signal, sharpening of a representation) can act on the representational hierarchy at all levels. Critically, this means that neither the operations, nor their putatively associated cognitive processes (e.g., recollection, familiarity, or perceptual priming) define how the pathway is carved up. Under this account, it is the hierarchy itself, rather than a set of separate memory systems, cognitive processes, or operations, that explains the organization of cognition in the ventral visual stream and MTL.

For a definition of recollection’s representational content, we appeal to a representational account of cognition. Representational accounts claim that the ventral visual stream and MTL form a pathway that represents increasingly high-dimensional conjunctions of stimulus features (Fig. 1). Early visual regions represent simple visual features individually, whereas later regions bring those features together into conjunctions, forming object parts, whole objects, and eventually combining objects with crossmodal information including context, time, and spatial location. Under this view, particular operations, such as pattern completion or generation of a memory strength signal, can occur anywhere in the pathway. What allows a brain region to contribute to a particular cognitive task is whether the brain region houses the representations that are necessary for the task. A relatively recent body of work provides support for this view by demonstrating that an important determinant of which MTL structures are engaged by a memory task is the content of the memory. Exactly what a participant is asked to learn and retrieve—objects or scenes, items or contexts, associations (or not)—influences which MTL structures, such as perirhinal cortex or hippocampus, are involved (Barense et al., 2005, 2010; Graham et al., 2006; Taylor et al., 2007; Poppenk et al., 2013; Berron et al., 2018; Newsome et al., 2018). This literature contrasts with studies supporting the more traditional view that it is mnemonic process that influences MTL recruitment (for review, see Eichenbaum et al., 2007; Suzuki, 2009). Reports that the hippocampus performs pattern separation of object stimuli may also pose a challenge for a representational content-based view (Bakker et al., 2008; Lacy et al., 2011; Yassa et al., 2011; Yassa and Stark, 2011), but a key question for future research is whether the contextual, temporal, and spatial information associated with those objects is an important aspect of what is being pattern separated.

Representational accounts suggest a way to characterize the content of a memory representation: the key property of a memory is its *dimensionality*, where high-dimensional memories are those containing arbitrary associations between complex, crossmodal, or spatial elements. This definition of representational content predicts differential engagement of brain regions: high-dimensional memories should engage hippocampus, whereas low-dimensional memories should engage pre-hippocampal neocortex. This definition also maps neatly onto the class of memories that are the typical targets of recollection: high dimensional memories corresponding to conjunctions of complex elements; e.g., items, context, and temporal information. That is, if recollection is a pattern completion-like retrieval process in which a cue prompts the recovery of information that was associated with the cue at encoding, then the label “high-dimensional” includes memories that are “recollect-able” and excludes memories that are not. Crucially, this definition is couched in terms of representational content only; it assumes nothing about the operations by which the memory may be accessed or retrieved.

As we described above when defining recollection’s operation, there are other definitions of representational content that might explain how distinct neural structures contribute to memory retrieval. We use this “dimensionality” definition as a test case, while acknowledging that it represents a small subset of the space of all possible hypotheses, as we discuss further below.

## Box 2. Testing the representational-hierarchical account of cognition

The representational-hierarchical account holds that the dimensionality of representations changes along a continuum (Fig. 1). One challenge in testing the account is deciding exactly which stimuli should engage each brain region. If a stimulus set does not engage a brain region as predicted, can the account always escape falsification by claiming that the stimulus level used was “not quite right” for the target region?

A critical property of the representational-hierarchical account is its assumption of a *continuum*: no one brain site is the “module” for conjunctions, rather, each region holds conjunctions of the stimulus attributes represented individually at the previous level in the hierarchy (Bussey and Saksida, 2002; Cowell et al., 2010b). Thus, the dimensionality of stimulus representations is relative: oriented lines are simpler than partial object fragments, which are simpler than faces and whole objects, which are simpler than scenes or spatial arrays of items. As dimensionality increases, the engagement and disengagement of successive brain regions should occur gradually. Because a clear-cut dichotomy between low- and high-dimensional is difficult to define, the representational-hierarchical account best makes predictions for the effects of a manipulation of dimensionality across a range of brain regions.

Moreover, whether a given brain region will be engaged in a task depends not just on the stimuli, but more broadly on the representational requirements of the task (Cowell et al., 2010b). Representational requirements are influenced by the particular properties of the stimulus set. For example, a set of face photographs including males and females of multiple races possessing highly distinct haircuts would be more easily discriminated than a set of faces in which all are females of the same race, with hair cropped from the images. The former can be discriminated on the basis of simple features (skin color, hair shape), whereas the latter can be distinguished only by subtle differences in overall configuration. Representational requirements are also influenced by task instructions. Tyler et al. (2004) showed that when participants name object images, the brain regions engaged by naming at the domain-level (living vs manmade) are different from those engaged by naming at the basic-level (e.g., rhinoceros or hammer). Presumably, when the response must be more specific, good performance requires more fine-grained, holistic representations.

**Figure 2.**
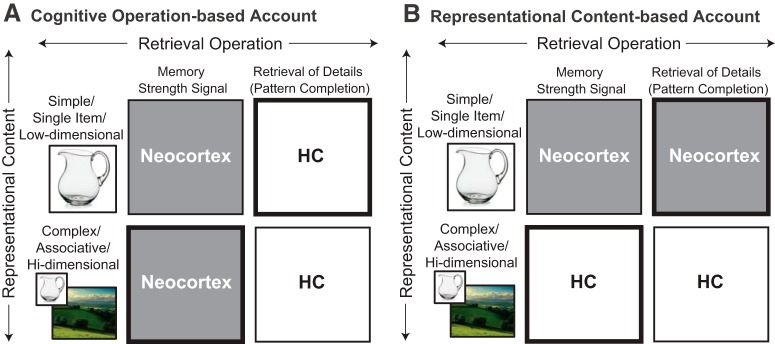
Two alternative accounts of the organization of memory in the ventral visual stream and MTL. ***A***, Under an account based on cognitive operations, brain regions are engaged according to retrieval operation; either a memory strength signal (Wickelgren and Norman, 1966; Kintsch, 1967), or retrieval of missing details from a partial cue (Montaldi and Mayes, 2010; Taylor and Henson, 2012). A memory strength signal can be provided by a representation that has been “sharpened” by encoding, whereas retrieval of missing details requires pattern completion between elements of a memory associated at encoding (Norman and O’Reilly, 2003). ***B***, Under an account based on representational content, brain regions are engaged according to the content of the retrieved memory; either lower-dimensional, single-item representations or higher-dimensional representations of arbitrary associations between complex elements. The bottom-left and top-right cells (thicker framing boxes) are more rarely tested and yet are critical for distinguishing the accounts.

Thus, testing the representational-hierarchical account requires examination of multiple brain regions via a systematic manipulation of representational requirements; these are determined by the stimulus class, the particular properties of the stimulus set and the task instructions. When testing the account, choosing two levels of representational dimensionality, “high” and “low”, provides a convenient experimental manipulation (Fig. 2). But the more general prediction is that increasing the dimensionality of the representational requirements is predicted to increase the recruitment of more anterior brain regions, and decrease the engagement of more posterior sites.

## Evaluating accounts of the neuroanatomical organization of retrieval against empirical data

Using these two definitions of retrieval operation and representational content, we can evaluate the alternative hypotheses for how memory is organized in the ventral visual-MTL pathway. First, we can examine whether it is the retrieval operation that determines the contribution of brain regions by asking: when the retrieval operation is varied and representational content held constant, are there systematic changes in which brain regions are engaged (Fig. 2*A*)? Next, we can pit this against the alternative hypothesis that representational content is the key determinant, by asking: when representational content is varied but retrieval operation held constant, does this reliably predict which brain regions contribute (Fig. 2*B*)?

If an operation-based hypothesis is correct, then pattern completion-like retrieval will always engage the hippocampus, regardless of memory content (Fig. 2*A*). If a content-based hypothesis is correct, then any memory supported by a high-dimensional representation that associates complex, crossmodal or spatial elements will engage hippocampus, regardless of the mechanism by which it is retrieved or accessed (Fig. 2*B*). In Figure 2, these hypotheses are laid out in a 2 × 2 matrix. For the top-left and bottom-right cells, evidence is already available. Many studies have revealed engagement of hippocampus during pattern completion-like retrieval of associative memories (Staresina et al., 2012, 2013; Hannula et al., 2013; Duncan et al., 2014; Tompary et al., 2016; Danker et al., 2017). Similarly, neocortical regions like perirhinal cortex are well established as critical for recognition memory of single items, which is assumed to be underpinned, at least in many animal tasks, by a memory strength signal (Staresina and Davachi, 2008; Hannula et al., 2013; Staresina et al., 2013). However, to distinguish between the two hypotheses, we need evidence for the two lesser-studied matrix cells: pattern completion-like retrieval of memories that are not high-dimensional (top-right) and high-dimensional memories retrieved by operations other than pattern completion (bottom-left).

### Pattern completion-like retrieval of memories that are not high-dimensional

**Figure 3.**
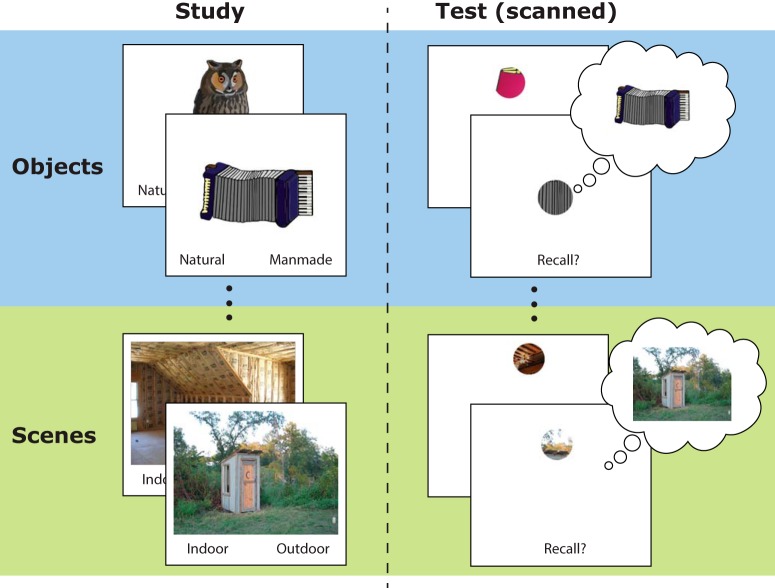
Experimental paradigm of Ross et al. (2018). Participants first studied whole objects and whole scenes. Later, in the scanner, memory was tested by presenting a patch of the studied image and asking participants to indicate with a button-press whether they recalled the corresponding object or scene. Recall was verified in a post-scan test in which participants again saw patches and this time typed the name of the recalled object/scene.

Ross et al. (2018) attempted to distinguish between operation-based and content-based accounts by testing pattern completion-like retrieval of both simple, single-item memories (Fig. 2, top right cell of matrix) and complex, associative memories (Fig. 2, bottom right cell). If an operation-based account is correct, then both should engage hippocampus (Fig. 2*A*), but if a representational account is correct, then retrieval of high-dimensional, associative memories should engage hippocampus whereas single-item retrieval should not (Fig. 2*B*). That is, representational accounts predict that if a task requires cued retrieval of a memory for which the association between the cue and the to-be-retrieved details resides outside of hippocampus (e.g., within-object associations, or bindings of simple features), then the task should not engage hippocampus (Shimamura, 2010; Cowell et al., 2010a). The key condition of Ross et al. (2018), was “object recall”, in which images of single objects were studied and memory was later cued with circular patches revealing part of an object, requiring pattern completion-like retrieval of the whole (Fig. 3, top). In an analogous control condition, participants studied and recalled complex scenes, which possess inherently associative content (Fig. 3, bottom). The key finding was that although patch-cued scene recall engaged hippocampus, patch-cued object recall did not; object recall instead engaged neocortical object-processing sites such as perirhinal and lateral occipital cortex. Thus, despite the requirement for pattern completion-like retrieval in both conditions, brain regions’ engagement was driven by memory content.

One possible interpretation is that, although hippocampus was not detectably activated by object recall, it still provided essential feedback for neocortical reinstatement of visual details; that is, hippocampus provided a pointer that triggered recall, without housing the representation itself (Teyler and DiScenna, 1985, 1986; Staresina et al., 2013; Bosch et al., 2014; Danker et al., 2017). But Ross et al. (2018) found evidence against this interpretation: effective connectivity analyses during both object and scene recall revealed increased flow of information from neocortex to hippocampus, not the reverse.

Other studies similarly point to the conclusion that pattern completion-like retrieval does not engage hippocampus when the memory is low-dimensional. Amano et al. (2016) induced learning of associations between low-level features in early visual cortex, and found that later presentation of one feature (“vertical”) led to retrieval of the other (“red”), as measured with fMRI. The effect was long-term, lasting 3–5 months, and yet control analyses indicated that it was not mediated by feedback from higher-level brain regions. Thus, the paradigm created long-term associative learning that enabled later part-cued retrieval, all within early visual cortex. Similarly, an fMRI study by Gorlin et al. (2012) provided evidence for pattern completion-like reinstatement of object details in visual cortex, using a paradigm that necessitated only low-dimensional (i.e., pre-hippocampal) representations. Participants identified Mooney images (degraded photographs) of objects, after having seen the photographs from which the images were created. A Mooney image thus provided a partial cue to memory for the photograph seen earlier. Classifier analyses revealed a slew of visual brain regions, substantially posterior to MTL, that reinstated information about a studied object (the photograph) on presentation of a partial cue (the Mooney image); a pattern completion-like retrieval operation.

Complementing these neuroimaging studies, Sadil et al. (2019) conducted a behavioral study of object memory and found that low-level, intra-object associations between the visual parts of an object can be learned and retrieved separately from associations between an object’s visual details and its name. Such part-to-part associations are required for the low-level pattern completion that we propose to underlie the findings of Ross et al. (2018) and Gorlin et al. (2012). Because Sadil et al. (2019) showed that these low-level associations influenced behavior independently of higher-level representations, this provides critical support for the idea that pattern completion-like retrieval can unfold at lower representational levels (Fig. 1).

Further evidence that pattern completion-like retrieval of low-dimensional memories does not depend on hippocampus comes from studies of amnesic patients. Warrington and Weiskrantz (1968, 1970) performed fragment completion tests in patients with damage to the hippocampal formation, using both line-drawn pictures and words. When given no instructions to supply information encountered at study, amnesic patients retrieved studied words or pictures from partial information (i.e., they completed fragmented pictures, fragmented words, or word stems by supplying studied items) at a rate comparable to controls. We interpret this as evidence that patients retrieved information via intra-item, part-to-whole associations, despite compromised hippocampal function. One of these studies, and a further three studies by Graf and coworkers, used another condition in which patients were explicitly asked to recognize or recall the same words or pictures. Under these circumstances, amnesics’ performance was markedly impaired (Warrington and Weiskrantz, 1970; Graf et al., 1984, 1985; Graf and Schacter, 1985). We suggest that, when asked to freely recall or to judge whether the words or pictures had appeared on a study list, participants were effectively asked to use the study context as a cue for retrieval, akin to asking, “Think of items that appeared in the study context”, or “Did this item appear in the study context?” Because the words and pictures had high pre-experimental familiarity, familiarity per se may have provided a weak discriminatory cue for recognition judgments, whereas the ability to retrieve a conjunction of item-in-study-context would have provided greater power to discriminate studied from unstudied items (Bird, 2017). Patients with compromised hippocampal function who lack item-context associations would thus be expected to show impaired performance on recognition and recall judgments that invoke the study context. We interpret the amnesic patients’ pattern of deficits on these tasks as evidence that pattern completion-like mechanisms for were intact for low-level (intra-item) associations, but impaired for high-level (item-context) associations.

As noted in Box 2, when testing the hypothesis that brain regions are engaged according to the dimensionality of the retrieved memory, it is important to consider not just the class of stimuli, but also the representational requirements of the task (Cowell et al., 2010b). To illustrate, we consider two fMRI studies that required pattern completion-like retrieval of ostensibly simple stimuli and yet showed hippocampal engagement. Bosch et al. (2014) created associations between simple stimuli: the pitch of an auditory tone and the orientation of a visual grating. Hippocampal activity was linked to the strength of cortical reinstatement in sensory cortex during cued recall. However, two features of this paradigm made learning and retrieval likely to be hippocampus-dependent, even under a dimensionality hypothesis (e.g., the representational-hierarchical account). First, the associated pairs were crossmodal, with one item visual and the other auditory, precluding an integrated representation within a single sensory cortex. Second, the retrieval task was complex, involving presentation of two auditory tones and a subsequent visual cue inducing participants to cast their mind back to the presentation order of the two tones, to determine which visual grating to retrieve (i.e., the task required relatively complex operations drawing on temporal information). According to a dimensionality hypothesis, to avoid engaging the hippocampus, the recall task must be solvable without engaging high-dimensional representations containing spatial relations and/or temporal information; a dimensionality hypothesis therefore clearly predicts hippocampal engagement for this task. Similarly, Rosenthal et al. (2016) reported increased V1-hippocampus coupling during a nonconscious recognition memory test in which participants were asked to recognize (i.e., judge as old/new) complex second-order sequences of visual stimuli defined by spatial location over time. The stimuli themselves were very simple: bright white circles appearing at two locations, presented separately to each eye, in sequences of 12 items. However, the discrimination of old from new at test relied on a representation of the conjunction of temporal position in sequence, visual field location, and left eye versus right eye presentation. According to a dimensionality hypothesis, such representations should engage hippocampal processing because they correspond to a crossmodal conjunction of spatial, temporal, and visual information (Fig. 1).

In sum, review of the literature reveals a number of studies that entailed a pattern completion retrieval operation applied to simple representational content. Together, these studies imply that humans can learn and retrieve associations residing at a lower level than the high-dimensional relations that depend on hippocampus, and that these associations can be learned and retrieved in neocortex without higher-level feedback. The critical point is this: when a memory does not involve high-dimensional associations between complex or spatial elements, pattern completion-like retrieval unfolds outside of hippocampus.

#### But are not all these findings just priming?

Absolutely, yes. Studies involving pattern completion-like retrieval of low-dimensional memories typically fall into the category of priming (Warrington and Weiskrantz, 1968). But, in fact, this convergence illustrates the central problem we hope to identify. At the outset of this review, we defined recollection, observing that it encompasses both a retrieval operation (pattern completion-like retrieval) and a class of representational content (high-dimensional, associative memories). Having decoupled the operation from the representational content and found examples of that operation applied to low-dimensional information, the resulting studies appear to be studies of priming. This seems to suggest that these two processes, recollection and priming, could be described in terms of common operations acting on different classes of representation. One might even argue that priming and recollection differ only in the representational content of the memories that are retrieved. But we stop short of making this argument: to claim that all instances of priming and recollection reflect a common retrieval operation would fail to reflect the multiplicity of ways in which the term “priming” is used.

A full treatment of priming is beyond the scope of this article, but “perceptual priming” is broadly defined as nonconscious influences of learning and retrieval on subsequent perceptual identification (Tulving and Schacter, 1990). However, specific characterizations of perceptual priming are extremely diverse: researchers have proposed that it is underpinned by pattern completion (Brunas et al., 1990); that it shares mechanisms with familiarity signaling (Jacoby and Whitehouse, 1989; Westerman et al., 2002; Huber et al., 2008); that it involves sharpening of representations in which irrelevant neural activity “drops out” (Wiggs and Martin, 1998); and that it increases the readiness of neural pathways such that a stimulus representation is subsequently evoked more quickly or efficiently (Henson et al., 2000; James and Gauthier, 2006). Clearly, the term perceptual priming encompasses a wide range of neural and behavioral phenomena.

Some have argued that priming phenomena can be grouped and understood under a multiple memory systems account, in which priming depends on a Perceptual Representation System (Tulving and Schacter, 1990), whereas declarative memory depends on MTL regions (Squire and Zola-Morgan, 1991; Squire and Dede, 2015). In these accounts, the term priming is used to refer not only to a set of behavioral phenomena, but also to the cognitive or perceptual process that is assumed to give rise to them. That is, a multiple memory systems view makes sense of the empirical evidence by acknowledging a multiplicity of learning modes (e.g., implicit, explicit), multiple classes of representational content (e.g., episodic, semantic, perceptual), and multiple memory processes (e.g., familiarity, recollection, priming), and then assigning them to distinct memory systems (Tulving and Schacter, 1990; Squire and Wixted, 2011; Squire and Dede, 2015). We agree that these multiplicities exist, but we do not believe that the processes attributed to the memory systems—recollection, familiarity, and priming—serve well as intermediate-level components of a memory theory (see Box 1). Priming, like recollection, is a behavioral phenomenon that we need to explain. When the term priming is used to refer to a mental process, even if it is characterized more specifically as perceptual priming, it fails to decompose the behavioral phenomenon associated with the process into a retrieval operation and a description of the representations that are retrieved.

But, despite the widespread use of the term priming to refer broadly to a mental process, many specific theories of priming-related phenomena have decomposed those phenomena into operations and representations. These theories, along with empirical tests of them, have revealed that, across a range of circumstances, priming may be underpinned by multiple operations (e.g., pattern completion, sharpening of representations, neural habituation) and multiple classes of representation (e.g., visual, auditory, conceptual; Hirshman et al., 1990; Schacter, 1992; Schacter and Church, 1992; Wiggs and Martin, 1998; Henson et al., 2000; Henson, 2003; Huber and O’Reilly, 2003; James and Gauthier, 2006 ). In our view, the careful theoretical and empirical work that has dissected priming phenomena illustrates that grouping such operations and representations under a common process label, priming, is misleading, and assigning them to a circumscribed memory system obscures their true nature. For example, priming may sometimes, but not always, involve the same operations as recollection. We believe that it is the underlying operations and representations—rather than the emergent, high-level phenomena of recollection and priming—that constitute the best intermediate-level components for building brain-based theories of cognition.

### Retrieving high-dimensional memories via operations other than pattern completion-like retrieval

Next, we examine the lower-left cell of the matrix (Fig. 2). When high-dimensional memories exert their influence on behavior via neurocomputational operations other than pattern completion, which brain regions are engaged? If the role of the hippocampus in memory performance is confined to the cued retrieval of missing details, it should not be engaged by other memory operations such as generation of a memory strength signal. Unambiguous examples of memory performance based on a memory strength signal alone are hard to find, because it is difficult to rule out the occurrence of cued recall even if the task does not require it: an item presented for a recognition judgment may automatically trigger thoughts of the encoding context. However, suggestions are provided by animal studies in which recognition memory is assessed via spontaneous behavior. Spontaneous recognition tasks exploit animals’ tendency to spend more time exploring a novel stimulus than a familiar one (Ennaceur and Delacour, 1988). The paradigm requires no explicit judgment, no decision criterion, and no part-cued recall; all the information required to discriminate novel from familiar is present in the test environment and behavior can be based on familiarity alone. (However, we acknowledge that lack of a requirement for pattern completion-like retrieval does not ensure that pattern completion is not occurring, and does not ensure that it is not contributing to memory performance.) Spontaneous recognition studies in rats with lesions imply a critical role for hippocampus, but only when retrieving or accessing complex, associative, or high-dimensional memories, for example, objects and spatial locations; objects and environmental context; objects and temporal context; or spatial arrangements of objects (Aggleton et al., 1999; Mumby et al., 2002; Eacott and Norman, 2004; Winters et al., 2004; Forwood et al., 2005; Good et al., 2007; Langston and Wood, 2010). Critically, the recognition of individual objects is not impaired by hippocampal lesions under most circumstances (Forwood et al., 2005; Jackson-Smith et al., 1993; Winters et al., 2004; but see, Clark et al., 2000, 2001; Zola et al., 2000). Thus, across animal studies in which familiarity is likely the principal driver of behavior, whether hippocampus is involved most often depends on the content—specifically, the dimensionality—of the memory.

Studies in human amnesics point to a similar conclusion. Cipolotti et al. (2006), Bird et al. (2007), and Hartley et al. (2007), tested recognition memory for faces, words, and topographical stimuli (buildings and landscapes) in individuals with focal hippocampal or more extensive MTL lesions. They found that hippocampus was involved in both recollective and familiarity processes for verbal and topographical information, but that, for faces, recollection and familiarity depend on extra-hippocampal regions. In related work, Mayes et al. (2002, 2004) reported a patient with selective hippocampal damage in whom recognition of items and intra-item associations was intact, but associative recognition was impaired. Similarly, several other studies have found that patients with selective hippocampal damage have poor recognition memory for previously-unknown buildings, words or scenes, but perform well at recognizing faces (Carlesimo et al., 2001; Taylor et al., 2007; Bird et al., 2008; Bird and Burgess, 2008; Smith et al., 2014). Corroborating this, Carlesimo et al. (2001) reported anecdotally that the patient seemed to experience a “sense of familiarity” for newly encountered laboratory staff but was unable to associate those faces with spatiotemporal context or with names. We suggest that verbal stimuli tend to recruit high-dimensional semantic/associative representations and scenes contain spatial relations that render them inherently high-dimensional, whereas unfamiliar faces constitute single perceptual items (Taylor et al., 2007; Bird, 2017), meaning these findings align with a dimensionality account of memory retrieval. However, Aly et al. (2010) reported mixed results regarding whether process (recollection vs familiarity) or stimulus material (faces vs words) better explains the involvement of hippocampus: estimates of both recollection and familiarity were reduced in MTL amnesics for words, but recollection and not familiarity was reduced in amnesia for faces, implying a role for both process and content in accounting for HC function.

We acknowledge that some of the foregoing studies had to make strong assumptions about the validity of a dual-process model of recognition memory to obtain estimates of familiarity and recollection (Cipolotti et al., 2006; Bird et al., 2007, 2008; Aly et al., 2010) and, in doing so, one revealed limitations to using this model for amnesic cases (Bird et al., 2008). Relatedly, other reviews of the behavioral recognition memory literature have concluded that the evidence for dual-processes in recognition memory is weak (Wixted, 2007; Dunn, 2008; Rotello, 2017). However, taking all of these studies together, including those that did not rely on assumptions of dual-processes in recognition memory, the balance of the evidence aligns with a content-based account in which the dimensionality of the memory, not the retrieval operation, determines hippocampal engagement.

## Alternative definitions of recollection: conscious awareness and intention

We have defined *recollection* as a pattern completion-like operation applied to certain content; high-dimensional memories defined by associations between complex elements. But this definition may be controversial, particularly the operation. Many theorists have emphasized not pattern completion, but intention, effort, or conscious awareness of retrieval (Hasher and Zacks, 1979; Tulving, 1985; Jacoby, 1991). So, do these factors better predict the involvement of hippocampus in memory performance?

It is known from amnesic cases that the hippocampus is important for explicit memory (Scoville and Milner, 1957; and documented widely thereafter). Thus, awareness-based accounts of memory would predict that retrieval without awareness does not involve hippocampus. But Henke et al. (2003) have shown using subliminal presentation that hippocampus does play a role in the implicit learning and retrieval of arbitrary associations (Degonda et al., 2005). Similarly, Hannula and Ranganath (2009) used eye-tracking to show that although conscious and nonconscious markers of relational memory retrieval can be dissociated, both engage hippocampus. In earlier work, Chun and Phelps (1999) reported a study of nonconscious associative memory in which hippocampal amnesics performing a visual search task were unable to learn and benefit from visuospatial context information that was acquired implicitly by controls. Further non-declarative memory tasks including categorization, perceptual learning and the statistical leaning of temporal regularities have all been found to engage hippocampus (Graham et al., 2006; Turk-Browne et al., 2009; Bornstein and Daw, 2012; Schapiro et al., 2012, 2013, 2014). Finally, in addition to awareness, others have examined the role of intention: Wang and Giovanello (2016) tested incidental versus intentional retrieval of learned word pairs in an fMRI study and found that intention had no effect on which MTL regions were recruited.

Together, these studies indicate that neither conscious awareness nor intent can explain the role of the hippocampus in memory. When high-dimensional memories are retrieved, the hippocampus is engaged regardless of conscious awareness, intention, or the specific retrieval operation.

## So does the notion of recollection still have a role to play?

Yes. Recollection is a salient, identifiable memory phenomenon, and our intention is not to deny its existence. Recollection can be disproportionately impaired by neurologic damage or disease (Yonelinas et al., 2002; Tsivilis et al., 2008; Vann et al., 2009), making it a phenomenon of great relevance and interest to clinicians: the inability to recollect can be devastating to patients. Moreover, to the layman, recollection is a salient phenomenon that is easy to report, rendering it useful as a dependent variable in memory studies. But when interpreting these reports, it is essential for memory scientists to understand that, in tests that ask participants to use or report additional information that they retrieve from memory, the factor determining whether a retrieval event is subjectively experienced as a recollection is the representational content of what is retrieved. That representational content is the kind of spatiotemporal, contextual information that is the currency of the hippocampus, e.g., a thought associated with an item at the time of encoding, or information such as which list an item appeared in.

We argue only that, as memory theorists, when using the term recollection, it is critical that we think carefully about both its representational content and its neurocomputational operation. To explain recollection, and why it is impaired by certain types of brain damage, we need to break it down into its component parts. In doing so, we reveal that the reason the neural substrate of recollection is always localized to a network involving the hippocampus is the representational content that recollection entails. Thus, using the term recollection to describe what a brain region does is misleading, because it obscures the important explanatory factor: in this case, representational content. Recollection is surely the province of the hippocampus, but by focusing on this process, a recollection-based explanation dwells at the wrong level of analysis and thereby fails to pinpoint the true currency of the hippocampus; namely, high-dimensional representations. Recollection, though critically dependent on hippocampus, is only one of many cognitive processes that exploit this underlying currency (Chun and Phelps, 1999; Lee et al., 2005a, 2012; Graham et al., 2006; Hannula and Ranganath, 2009; Aly et al., 2013).

Therefore, our recommendation for the term recollection is not that it be removed from memory researchers’ vocabulary altogether, but that it be acknowledged for what it is: a qualitative, behavioral phenomenon that adds richness to our high-level description of human memory retrieval, not a mechanistic component of a memory theory.

## Defining the space of hypotheses to be explored

A difficult but important challenge in asking whether operations or representations best explain neuroanatomical organization is deciding which operations and which definitions of representational content are the best candidates for exploration. In the 2 × 2 matrix of Figure 2, we consider only two operations and two classes of representation. It is possible that we explored the wrong set of hypotheses for the operation axis, giving representations an unfair advantage. Perhaps there were not 2 but 4, or even 10 plausible models along each axis. And, even if these were appropriate candidate hypotheses for systems-level organization (Churchland and Sejnowski, 1988), we may need to consider alternative definitions of operation and representation for organization at a finer scale. For example, among subregions of the hippocampus, it has been proposed that CA1 computes a comparator operation whereas CA3 supports pattern completion (Hasselmo and Schnell, 1994; Vinogradova, 2001)

In general, if we consider only a subset of the model space, we can conclude only that the model favored by the evidence is the best of those considered, not that it is the true underlying model. In this review, we have examined a very limited set of hypotheses: whether recruitment of the ventral visual-MTL pathway during declarative memory retrieval is better explained by the operations associated with recollection and familiarity, or by the dimensionality of a retrieved memory. We believe that examining this small model space is nonetheless valuable because the outcome has important implications for theories and empirical studies of memory, many of which tacitly or explicitly assume a distinction between recollection and familiarity. Although the favored hypothesis—the dimensionality of representations—is assuredly an oversimplification, or may be incorrect, the exercise of comparing the two hypotheses has provided a recommendation for narrowing the field of possible hypotheses and might thereby accelerate progress toward the best possible model. Reaching this conclusion does not entail that we stop investigating other possible mnemonic operations, nor rule out the possibility that operations could sometimes trump representations in the neuroanatomical organization of cognition.

## How does the operations and representations approach apply to encoding?

In this review we examine retrieval, but considerable empirical work has examined the neural basis of encoding. A full treatment of this literature is beyond the present scope, but many encoding studies suggest that, at a relatively coarse-grained neuroanatomical resolution (i.e., comparing hippocampus, MTL neocortex, and ventral temporal cortex), the organization of encoding echoes that of retrieval. That is, the representational content of the encoded memory seems to determine which region is engaged at retrieval (Sperling et al., 2001, 2003; Davachi et al., 2003; Brassen et al., 2006; Chua et al., 2007; Hayes et al., 2007; Awipi and Davachi, 2008; Staresina and Davachi, 2008; Mei et al., 2010). The details of the operations employed at encoding are also beyond the scope of the present review, and more work is required to specify them precisely. But in our view, the representations laid down at encoding are those retrieved by pattern completion at retrieval, and so although the representations themselves are agnostic to encoding and retrieval, the two sets of operations are likely to be intimately related.

It may be that certain operations at encoding are specialized to create certain representations, e.g., “contextual binding” may be required to create high-dimensional representations in hippocampus (Eichenbaum et al., 1994; Henke et al., 1997; LaBar and Phelps, 2005). However, we still prefer to separate the word “contextual” from the word “binding”, because the first refers to content and the second to an operation such as associative learning. Associative learning can occur for features, too, and to the extent that this operation contributes to both feature-binding and contextual-binding we prefer to describe it with a content-neutral label, to reveal commonalities between operations occurring in different parts of the brain.

## Applying the operations and representations approach beyond long-term memory retrieval

Our framework advocates the decoupling of operations from representations. When we applied it to the question of memory retrieval, we concluded that a single operation, pattern completion, can act on multiple classes of representations, housed across multiple brain regions. This analysis draws mechanistic parallels between recollection and priming, and helps resolve the debate over the systems-level neuroanatomical organization of memory. But we believe that the “operations and representations” approach has explanatory utility beyond long-term memory. There are many examples, in psychology and neuroscience, not only of one operation acting on multiple classes of representation, but also of one class of representations being acted on by multiple operations.

Indeed, the representational-hierarchical account originated in an explanation of how perirhinal cortex contains a single class of representations—objects—but contributes to two cognitive functions: memory and visual perception (Bussey et al., 2002, 2003; Bussey and Saksida, 2002; Cowell et al., 2006, 2010b; Sadil and Cowell, 2017). Under this account, the perirhinal cortex contributes to many different tasks involving objects by virtue of the many operations that can act on its representations. This was supported empirically by demonstrations that perirhinal cortex is in involved in perceptual oddity judgements (Buckley et al., 2001; Barense et al., 2007, 2011; Bartko et al., 2007; Lee et al., 2008), recognition memory (Meunier et al., 1993; Eacott et al., 1994; Winters et al., 2004), visual discrimination (Barense et al., 2005, 2012; Sadil and Cowell, 2017), and the representation of conceptual object knowledge (Barense et al., 2010, 2011; Clarke and Tyler, 2014; Martin et al., 2018). A similar story has unfolded in hippocampus: many studies have now demonstrated that the high-dimensional representations in hippocampus are acted on by many different operations in the service of many different tasks (Aly and Turk-Browne, 2018). The list of cognitive functions dependent on the hippocampus now includes the perceptual discrimination of visual stimuli (Lee et al., 2005a,b, 2006, 2007); attention (Aly and Turk-Browne, 2016a,b); decision-making (Shohamy and Daw, 2015); scene construction, regardless of the past or future status of the event (Palombo et al., 2018); associative aspects of language tasks (Duff and Brown-Schmidt, 2012); supplying the relational representations that underlie creativity (Duff et al., 2013); providing a high-dimensional social cognitive map (Schafer and Schiller, 2018); and imagination and prediction (Buckner, 2010). Thus, the hippocampus may house only one class of representation, high-dimensional representations, on which many operations act. But these operations may not be unique to hippocampus. Operations like pattern completion or the generation of a prediction error (Friston, 2010) are replicated across many cortical sites. Our hope is that explicit identification of the operations and representations underlying cognition will draw parallels between disparate tasks, helping to identify key properties of neural mechanisms that are unified or divergent across tasks and across brain regions.

## Box 3. Questions for future research

•  To what extent do representations (vs operations) explain the neuroanatomical organization of other cognitive functions, such as working memory, and other brain regions, such as prefrontal cortex?
•  To what extent do representations (vs operations) explain the neuroanatomical organization of cognitive function at more fine-grained biological scales, such as between subregions of the hippocampus (DG, CA1, CA3)?
•  Besides vision, do other sensory systems (e.g., audition, gustation) follow a hierarchical organization in which the dimensionality of representations can help to explain the contribution of each stage to perception and cognition?
•  Do the representations and operations computed by particular brain regions remain constant over relatively short time frames, or can they change dynamically during task performance?
•  Will the identification of the operations underlying performance of diverse tasks draw parallels between disparate literatures (cf. the parallel between recollection and priming drawn here)?
•  The 2 × 2 hypothesis space of Figure 2 captures only a small subset of all possible models. What other operations and representational properties should be considered in future work?
•  Can we build computational models with explicit mechanisms that unify performance across tasks via shared operations or shared representations?

## Conclusions

In theories of the neural basis of memory, cognitive processes like recollection, familiarity, and priming have played a prominent role. But such processes are often hybrid concepts that invoke both a neurocomputational operation and a class of representational content. Recollection is one such hybrid, in which the operation is pattern completion and the representational content is high-dimensional memories comprising associations of complex elements. Because a full theory of memory must specify both operations and representations, a label like recollection, which conflates the two, is couched at the wrong level for characterizing brain function. We introduce a novel framework for understanding the functional division of labor in cortex, in which we ask whether the neuroanatomical organization of memory is better described by operations or by representational content (Fig. 2). The data suggest that when the content of a memory is high-dimensional, retrieval depends on the hippocampus, even when the retrieval operation is not pattern completion (Fig. 2*B*, bottom left cell). Conversely, when a memory is lower-dimensional, it is retrieved outside of hippocampus, even when it is retrieved by a pattern completion-like operation (Fig. 2*B*, top right cell). For memory performance, the brain appears to be carved up according to representational content, not retrieval operations.

This conclusion has important implications for memory models. There has long been a debate between dual-process and content-based theories (Yonelinas, 2001; Yonelinas AP (2002); Barense et al., 2005; Aggleton and Brown, 2006; Cowell et al., 2006; Graham et al., 2006; Lee et al., 2006; Taylor et al., 2007). Recent models appear to resolve this debate by accounting for the contributions of distinct brain regions to memory by invoking both the representational content of a memory and a recollection process, and emphasizing that recollection applies to a particular class of representational content (Diana et al., 2007; Mayes et al., 2007; Frank et al., 2008; Yonelinas, 2013; Sadeh et al., 2014). But we have argued that to properly specify the neural mechanisms of memory retrieval, we must decompose recollection into an operation and a representation, and examine the influence of each in isolation. Taking this route, the debate between process-based and representational content-based models is resolved, not via theories that emphasize both recollection and its associated representational content, but more parsimoniously, in favor of representational accounts (Graham et al., 2010; Ranganath, 2010; Cowell et al., 2010a).

One potential interpretation of our view is that the neural mechanisms of retrieval (whether under the guise of priming or recollection) are implemented identically all over the brain. After all, we claim that pattern completion-like retrieval occurs in both visual cortex and hippocampus. When V1 neurons mediate retrieval of a color from an orientation, do they use the same mechanisms (e.g., cell types, neurotransmitters, and synaptic signals) as hippocampal neurons reinstating an episodic event from a context cue? If this were true, why would the hippocampus have such radically different cytoarchitecture from sensory neocortex (Insausti et al., 2017)? We resolve this contradiction by considering the level of explanation. We do not claim that low-level (e.g., molecular, synaptic) mechanisms for cued retrieval of associations in visual cortex are identical to those for hippocampal recall of complex events. A key difference is the dimensionality of the representations: very low for V1, very high for hippocampus. These representations may require, in V1, only simple local circuits, but in hippocampus, specialized architecture to create separate, unique instances of high-dimensional stimuli that share features. Nevertheless, although the specifics of how pattern completion acts on these different representations may diverge at the neural level, at the level of cognition (or at Marr’s algorithmic level), the pattern completion operation is analogous.

Moreover, we do not claim that cognitive operations are irrelevant to the brain-basis of cognition. Any satisfying memory theory must specify the mental operations that act on neural representations to give rise to phenomena such as recollection and familiarity (see Box 1). Neither do we claim that representations always trump representations, in the neuroanatomical organization of cognition. For questions other than the one we address here, i.e., other than the coarse-grained organization of long-term declarative memory retrieval, it may be that operations are more crucial than representations for explaining the role of distinct regions. For example, at a fine-grained scale within the hippocampus, the roles of the dentate gyrus (DG) and CA3 subfields may be in part explained by contrasting operations: the DG appears critical for creating orthogonal representations for efficient storage, a pattern separation operation performed at encoding, whereas CA3 appears to be important for generalization from partial or noisy inputs to a learned representation, a pattern completion operation at the time of retrieval (Leutgeb et al., 2007; Leutgeb and Leutgeb, 2007; Neunuebel and Knierim, 2014). Another example is the prefrontal cortex (PFC): whereas some accounts of how PFC subregions differentially contribute to working memory are representational, or “material-specific” (Goldman-Rakic, 1987; Levy and Goldman-Rakic, 2000), other successful models have instead claimed that subregions of PFC can be characterized by their operations, such as maintenance versus manipulation of memory representations (D’Esposito et al., 2000; Petrides, 2000; Wagner et al., 2001; Curtis and D’Esposito, 2003). For any given research question within cognitive neuroscience, we harbor no a priori bias in favor of representations over operations. Our central message is simply that high-level phenomena such as recollection, familiarity, and priming—which at best obscure and at worst conflate representations and operations—are not the right labels for distinguishing the roles of neuroanatomical structures in cognition.

Thus, our arguments are limited, but we hope that they will help reshape how research questions are framed in cognitive neuroscience. Any reader who hopes to find in this review a theory that can explain the distinction between implicit versus explicit memory, short-term versus long-term memory, or visual versus semantic versus episodic memory will be disappointed. Our goal is not to explain those distinctions, nor to deny that they exist. Our goal is to argue that these introspectively salient categories are not the best way to explain how brain structures or systems contribute to cognition. In the spirit of Hasson et al. (2015), who proposed that memories of all durations emerge from, and interact across, a continuous hierarchy of memory timescales in the brain, we emphasize the importance of a continuous hierarchy of memory representations. Like Hasson et al. (2015), we favor distributed memory over compartmentalized memory systems. There are undoubtedly different types of memory and a multiplicity of neural mechanisms for learning and retrieval. But the different memory types do not map neatly onto distinct neural operations and the operations are not tied exclusively to distinct neural substrates. Research efforts that attempt to map introspectively-defined, phenomenological processes onto neuroanatomical targets may place the emphasis on the wrong theoretical concept, slowing the progress of understanding (Anderson, 2011). Perhaps, in our continuing game of pin the tail on the donkey, it is time to change out the tail, replacing processes with descriptions of representational content and neurocomputational operations. Challenges for future research will include better characterizing these representations and more precisely specifying the operations that act on them to produce key behavioral phenomena.

## Synthesis

Reviewing Editor: Mark Baxter, Mount Sinai School of Medicine Decisions are customarily a result of the Reviewing Editor and the peer reviewers coming together and discussing their recommendations until a consensus is reached. When revisions are invited, a fact-based synthesis statement explaining their decision and outlining what is needed to prepare a revision will be listed below. The following reviewer(s) agreed to reveal their identity: Vishnu Murty.

The reviewers were very enthusiastic about the paper, but had suggestions for broadening the scope and clarifying some points in order to further increase its impact.

1. One key challenge of assessing whether operations or representations explain a brain region's contribution to cognition is in defining which relevant operations and representations to explore. In fact, this is of fundamental importance for this approach to have traction. Do we have a good enough understanding of those axes (operations, representations)? I like the idea of having a 2 x 2 matrix of operations by representations, but it would help to acknowledge that there are many more possibilities on either axis (e.g., it could end up being a 10 x 10 matrix [or larger!] for all we know!). The authors do mention that pattern completion isn't universally accepted as the key mechanism for recollection. But what about other mnemonic processes, such as the comparator function of CA1? The idea is that CA1 can compare expectations (from CA3 pattern completion) to reality (from ERc input) and output a signal related to match/mismatch. Pattern completion is certainly a subset of this process -- but it's only part of it. How do we arrive at the right components of each axis (e.g., the relevant operations), and how do we know we have them all? It would be useful to discuss other memory-related operations such as predictions errors, expectancy violations, and contextual binding. I think the more operations that can be tested, the more likely the authors conclusions that representations are the underlying feature become stronger.

2. Continuing on point #1: As a second problem, how do we specify the representational axis? What counts as high dimensional, complex, and associative? Objects are low dimensional compared to object-scene associations, but high dimensional compared to oriented lines. Are fractals high or low dimensional? Faces are argued to be low dimensional in this paper, but one could argue they are high dimensional - although processed holistically, they contain many features and relations, and are certainly higher dimensional than the parts of which they are made. The danger is, if there is not a clearly specified definition of what constitutes high vs low dimensional, then we can fall into the trap of saying stimulus X must be high dimensional because it recruited the hippocampus; stimulus Y must be low dimensional because it did not. Can the authors specify, explicitly and unambiguously, how one knows when something is high vs low dimensional? Or perhaps this dichotomy can be abandoned, with the prediction that gradually increasing dimensionality will be associated with monotonic increases in hippocampal engagement (for example)?

3. The broad concept of recollection vs familiarity has been addressed both from an encoding and retrieval standpoint. However, the current manuscript tends to focus mostly on retrieval (especially given the emphasis on pattern separation). It would be nice to discuss how their perspective relates to encoding. In other words, what putative operations may be driving memory success across these regions during encoding, or is it all about representation in the encoding context.

4. The authors come down on the side of a representational account, but briefly acknowledge that for other domains, operations may be a better descriptor of brain regions' functions. They also briefly mention that operations are a key differentiating factor across hippocampal subfields. The paper might benefit from a more detailed elaboration on this. Even within memory, representations might be the best account of brain region X's function (e.g., hippocampus), but operations might be a better descriptor of brain region Y's function. Do the authors believe that memory is better explained by a representational account, period, or are operations more important than representations in some brain regions (e.g., parietal cortex? prefrontal cortex? striatum?) Do differences in the neural architecture of different brain regions (i.e., cell types, neuromodulatory receptors, layers of cortex) change the possibility of what operations may be performed at each level. This may be particularly relevant for pattern separation. Are the authors suggestions that the mechanistic operation of pattern separation is identical in PRC versus hippocampus, despite the drastic differences in the functional machinery located in each of these areas. If these operations were different, there is a light concern that “pattern separation” has become another functional label. Similarly, how does this idea hold up against theories of working memory which suggest that the lateral PFC only holds the operations of working memory maintenance/updating/manipulation, while also the representations are stored in perceptual areas (i.e., Desposito, Postle, Sreenivasan).

5. On p. 8, the authors cite work that MTL involvement in memory depends on the content to be remembered. There is certainly a lot of evidence for this. But there is also a lot of evidence that the hippocampus/parahippocampal cortex vs perirhinal cortex play roles in recollection vs familiarity, respectively, that extend across many stimulus materials (e.g., Eichenbaum, Yonelinas, & Ranganath, 2007, Annu Rev Neurosci). Contradictory evidence should be cited: not all findings seem to concord with a representational account.

6. The authors cite work showing that low-dimensional pattern completion-like retrieval does not engage the hippocampus. But what about Bosch, Jehee, Fernández, & Doeller, 2014, J Neurosci, and Rosenthal, Andrews, Antoniades, Kennard, & Soto, 2016, Current Biology? And Kok, Rait, & Turk-Browne, 2019, bioRxiv? Do those contradict this viewpoint? If not, why are the stimuli in those studies not considered ‘low-dimensional’?

7. The authors cite some neuroimaging work showing that low-dimensional pattern completion-like retrieval does not engage the hippocampus. They then conclude that “...these associations can be learned and retrieved in neocortex without higher-level feedback”. However, fMRI can never tell you that: aside from this claim resting on null findings, fMRI is only correlational, and can never tell us whether a brain region is necessary for a cognitive function or not. To make that claim, there would have to be evidence that, without a functioning hippocampus, pattern completion-like retrieval can still occur for low dimensional stimuli. Can the authors discuss evidence for this? Moreover, research from the Stark group (and its descendents) have provided evidence of object based pattern separation in the hippocampus, which goes against the authors' conclusions. The authors need to incorporate these findings into the manuscript.

8. The term ‘recollection’ does seem to be used in an operation-based way by some researchers. For example, in the DPSD model, recollection has been considered a high threshold signal, such that some amount of evidence triggers the retrieval of detailed contextual information, but evidence below that amount does not lead to any retrieval at all. Does that count as an operation? If not, why not? Why does the phenomenon of pattern completion ‘count’ as an operation more than this definition? Relatedly, many cognitive neuroscientists have abandoned Recollection/Familiarity as a framework and have used other functional labels such as item/relational (i.e., Cohen, Davachi) or Items/Items in Context (i.e., Diana, Ranganath). How does the authors' framework of comparing representations/processes go above and beyond what we have garnered from using these alternative functional labels?

9. The claim this paper makes in not assigning phenomenological processes to distinct brain regions is much broader than the memory literature. For example, as the authors know quite well, there is no clear separation of perception vs memory in the brain. Can the authors briefly comment on the utility of their perspective in explaining findings outside of memory as well, e.g., the efficacy of the representational account in explaining the role of MTL structures in perception, attention, etc? That can increase the impact of the paper: although it is a paper about memory, it applies much more broadly, and indeed this framework has proven useful for understanding MTL contributions to perception, attention, and beyond. Can the authors give other examples, from elsewhere in psychology/neuroscience, where a given operation is applied to multiple representations, and a given representation acted on by multiple operations? It might spur more research of this kind outside of the memory field.

Other suggestions:

1. Can the authors add a box of future directions or predictions? It would be great to get a sense of which specific, novel predictions this theory makes that will push new research directions.

2. On p. 13, the authors describe a task that can be accomplished based on familiarity alone, yet recruited the hippocampus when the information was high dimensional. Just because a task only requires familiarity does not mean animals are only using familiarity.

3. On p. 13, the authors describe work showing that both recollection and familiarity for faces depend on extra-hippocampal regions. But see Aly, Knight, & Yonelinas, 2010, Neuropsychologia. Another place where it seems necessary to acknowledge contradictory results.

4. p. 4: “representations that give rise them” > “representations that give rise to them”

5. p. 15: “operation is pattern completion the representational content” > “operation is pattern completion, and the representational content”

6. p. 17: “high level phenomena such recollection” > “high level phenomena such as recollection”
